# Therapeutic iloprost for the treatment of acute respiratory distress syndrome (ARDS) (the ThIlo trial): a prospective, randomized, multicenter phase II study

**DOI:** 10.1186/s13063-020-4163-0

**Published:** 2020-03-04

**Authors:** Helene Haeberle, Stefanie Prohaska, Peter Martus, Andreas Straub, Alexander Zarbock, Gernot Marx, Manola Zago, Martin Giera, Michael Koeppen, Peter Rosenberger

**Affiliations:** 1 Department of Anesthesiology and Intensive Care Medicine, University Hospital, Universitätsklinikum Tübingen, Universitätsklinikum Tübingen, Hoppe-Seyler-Straße 3, 72076 Tübingen, Germany; 2 Institute for Clinical Epidemiology und Applied Biostatistics, Eberhard Karls University, Tübingen, Germany; 3 Department of Anesthesiology, Intensive Care Therapy and Pain Medicine, University Hospital Münster, Münster, Germany; 4 Department of Intensive Care Medicine and Intermediate Care, University Hospital RWTH Aachen, Medical Faculty RWTH Aachen University, Aachen, Germany; 5 Center for Clinical Studies, University Hospital, Tübingen, Germany; 6 Center for Proteomics and Metabolomics, Leiden University Medical Center (LUMC), Leiden, The Netherlands

**Keywords:** ARDS, Iloprost, Inflammation, Intensive care, Ventilation

## Abstract

**Background:**

Acute respiratory distress syndrome (ARDS) is caused by rapid-onset (within hours) acute inflammatory processes in lung tissue, and it is a life-threatening condition with high mortality. The treatment of ARDS to date is focused on the prevention of further iatrogenic damage of the lung rather than the treatment of the initial inflammatory process. Several preclinical studies have revealed a beneficial effect of iloprost on the control of pulmonary inflammation, and in a small number of patients with ARDS, iloprost treatment resulted in improved oxygenation. Therefore, we plan to conduct a large multicenter trial to evaluate the effect of iloprost on ARDS.

**Methods:**

The Therapeutic Iloprost during ARDS trial (ThIlo trial) is a multicenter, randomized, single blinded, clinical phase II trial assessing the efficacy of inhaled iloprost for the prevention of the development and progression of ARDS in critically ill patients. One hundred fifty critically ill patients suffering from acute ARDS will be treated either by nebulized iloprost or NaCl 0.9% for 5 days. Blood samples will be drawn at defined time points to elucidate the serum levels of iloprost and inflammatory markers during treatment. Mechanical ventilation will be standardized. In follow-up visits at days 28 and 90 as well as 6 months after enrollment, functional status according to the Barthel Index and a health care-related questionnaire, and frailty (Vulnerable Elders Survey) will be evaluated. The primary endpoint is the improvement of oxygenation, defined as the ratio of PaO_2_/FiO_2_. Secondary endpoints include 90-day all-cause mortality, Sequential Organ Failure Assessment scores during the study period up to day 90, the duration of mechanical ventilation, the length of intensive care unit (ICU) stay, ventilator-associated pneumonia, delirium, ICU-acquired weakness, and discharge localization. The study will be conducted in three university ARDS centers in Germany.

**Discussion:**

The results of the ThIlo trial will highlight the anti-inflammatory effect of iloprost on early inflammatory processes during ARDS, resulting in the improvement of outcome parameters in patients with ARDS.

**Trial registration:**

EUDRA-CT: 2016-003168-37. Registered on 12 April 2017. ClinicalTrials.gov: NCT03111212. Registered on 4 June 2017.

## Background

Acute respiratory distress syndrome (ARDS) is defined as pulmonary compromise with bilateral pulmonary infiltrates associated with moderate to severe hypoxemia [1]. The public health impact of ARDS is considerable, and it is estimated that approximately 75,000 cases of ARDS occur annually in Germany. The estimated mortality ranges from 26 to 51% and depends on the severity of the associated hypoxemia [2]. Patients surviving ARDS treatment also show reduced functional capacity in their everyday life following hospitalization [3, 4]. Therefore, there is a pressing need to develop further ARDS treatment strategies with a view to ultimately improving patient outcomes.

The bilateral pulmonary infiltrates that can be identified on chest radiography reflect the diffuse inflammatory changes within the lung that are caused by acute inflammation within the pulmonary tissue and the alveolar space. The initial inflammatory process is induced by the activation of the innate immune response by the binding of microbial products (pathogen-associated molecular patterns [PAMPs]) or cell injury-associated endogenous molecules (danger-associated molecular patterns [DAMPs]) to pattern recognition receptors (PRRs). Therefore, the common causes of ARDS are trauma, sepsis, pneumonia, blood transfusion, or aspiration into the lungs. After the initial activation of the innate immune response, innate immune effector mechanisms, such as the formation of neutrophil extracellular traps (NETs), are activated, which further aggravate the alveolar injury [5]. The resulting increased permeability of the microvascular barrier results in the extravascular accumulation of protein-rich fluid that accumulates within the alveolar space. The increased permeability is also linked to the transfer of leukocytes (mostly neutrophil granulocytes) and erythrocytes into the alveolar space in ARDS, as well as to the presence of proinflammatory-regulated cytokines that increase the inflammatory burden within the lung [5]. As a result, dysregulated inflammation, the accumulation of leukocytes and platelets, and altered permeability of alveolar barriers remain the central pathophysiologic problems in ARDS [5, 6].

The treatment of ARDS to date is focused on the prevention of further iatrogenic damage of the lung through lung-protective mechanical ventilation, neuromuscular blockade, and conservative fluid management [7]. Recent clinical trials have focused on the role of ventilation strategies in the prevention or treatment of ARDS using noninvasive ventilation devices or prone positioning [8, 9]. Although these strategies have shown a positive effect on patient oxygenation and symptoms, they do not interfere with the underlying pathophysiological changes of ARDS. Several interventions have tried to use a potential anti-inflammatory strategy for the treatment of the existing intra-alveolar inflammation or to intervene in the development of intra-alveolar inflammation. For this, patients were treated with aspirin, simvastatin, and surfactant, but the tested treatments failed and did not have any significant effect [10–12].

### Iloprost

Considerable evidence in preclinical models shows that the use of iloprost for the treatment of ARDS and pulmonary inflammation might be of significant benefit. In small animal models, investigators showed that iloprost improves endothelial barrier function and reduces the detrimental signs of pulmonary edema [13]. It also reduces the pulmonary sequestration of leukocytes and platelets, which is a central disease mechanism underlying the development of ARDS [14]. This evidence could be transferred into different models of lung injury, showing positive evidence for the reduction of pulmonary inflammation in a pressure-induced model of lung injury [15]. The anti-inflammatory effect was attributed to the cyclooxygenase-2 (COX-2) system and the involvement of lipoxin A4 [16]. Ras-related protein 1 (RAP-1) might also be involved in the protective role of iloprost [17]. This positive anti-inflammatory effect of iloprost on the pulmonary tissue was also demonstrated in several models of ischemia-reperfusion (IR) injury. Furthermore, IR injury can also result in ARDS and pulmonary failure. Iloprost was able to reduce this pulmonary compromise in several preclinical studies [18–21]. The anti-inflammatory effect of iloprost was also shown in large animal models of lung injury using porcine models of ARDS [22–24]. Here, again, iloprost showed an anti-inflammatory effect. In addition, the shunt fraction could be reduced, which resulted in improved oxygenation and improved pulmonary dynamics, which is essential for the reinstitution of spontaneous ventilation during and following ARDS [22, 23, 25–27]. This shows that the preclinical data identified a beneficial effect of iloprost on ARDS. So far, only one study on inhaled iloprost in adult patients with ARDS has been conducted, although an application of inhaled iloprost is noted in the guidelines of the Association of the Scientific Medical Societies (AWMF) for the treatment of ARDS [28]. The AWMF guidelines indicate that the use of ARDS can be considered, especially in patients with severe ARDS who are mechanically ventilated and not self-consenting [7].

## Methods/design

### Study design

ThIlo is a multicenter, randomized, single blinded clinical phase II trial assessing the efficacy of inhaled iloprost in the development and progression of ARDS in critically ill patients. Based on the risk of pulmonary hemorrhage, which is very rare—especially in patients with ARDS—the study medication was unblended. For safety reasons, after treatment of 100 patients (day 28 after last dose investigational medicinal product [IMP] Patient 100) within the study, an interim analysis for an increased risk for pulmonary hemorrhage ≥ grade III according to Common Terminology (Toxicity) Criteria for Adverse Events (CTCAE) Version 5.0 in the treatment (iloprost) arm will be performed and the results discussed with the Data and Safety Monitoring Board (DSMB). The study was approved by the local ethics committee (University of Tübingen, Germany) on June 4^th^, 2019 and by BfArM (Bundesinstitut für Arzneimittel und Medizinprodukte, Bonn, Germany, EU/1/03/255/001) on the 14th of March 2019. The trial is registered at EUDRA-CT (2016-003168-37) and at ClinicalTrials.gov (NCT03111212). The Standard Protocol Items: Recommendations for Interventional Trials (SPIRIT) checklist is provided as Additional file 2.

### Population

The target population for this clinical trial is adult critically ill patients with ARDS. Patients will be included in the trial if they present with ARDS as defined by the Berlin definition (Table 1 and [1]) and meet the inclusion criteria. The trial population will consist of both sexes. One hundred fifty intensive care patients with ARDS will be included in the study at the Department of Anesthesiology, Eberhard Karls University Tübingen, Germany; the Department of Intensive Care and Intermediate Care, University Hospital RWTH Aachen, Germany; and the Department of Anesthesiology, University Hospital Münster (UKM), Münster, Germany.

**Table 1:** Definition of ARDS

Mild ARDS	Moderate ARDS	Severe ARDS
200 mmHg < PaO_2_/FiO_2_ ≤ 300 mmHg PEEP ≥5 cmH_2_O	100 mmHg < PaO_2_/FiO_2_ ≤ 200 mmHg PEEP ≥5 cmH_2_O	PaO_2_/FiO_2_ ≤ 100 mmHg PEEP ≥5 cmH_2_O

### Inclusion criteria

Patients meeting the following criteria will be included: age ≥ 18 years, PaO_2_/FiO_2_ ≤ 300, bilateral infiltrates consistent with pulmonary edema on frontal chest radiograph, need for positive pressure ventilation via an endotracheal tube or noninvasive ventilation and no clinical signs of left atrial hypertension detected via echocardiography, or if measured, a pulmonary arterial wedge pressure (PAWP) less than or equal to 18 mmHg. The term “acute onset” is defined as follows: the durations of the hypoxemia criterion and the chest radiograph criterion must be ≤48 h at the time of randomization. Patients must be enrolled within 48 h of ARDS onset and no later than 7 days from the initiation of mechanical ventilation.

### Exclusion criteria

The exclusion criteria are defined as follows: subject age < 18 years; time interval more than 7 days since the initiation of mechanical ventilation; more than 48 h since the onset of ARDS; patient, surrogate, or physician not committed to full intensive care support; positive pregnancy test at the time of screening; and contraindications against iloprost. These are defined as conditions in which the effects of iloprost on platelets might increase the risk of hemorrhage (e.g., active peptic ulcers, trauma, intracranial hemorrhage), severe coronary heart disease, myocardial infarction (within the last 6 months), decompensated heart failure, severe arrhythmias, unstable angina pectoris, pulmonary arterial hypertension caused by the occlusion of pulmonary veins, cerebrovascular events (e.g., transient ischemic attack, stroke) within the last 3 months, and congenital or acquired valvular defects with clinically relevant myocardial function disorders not related to pulmonary hypertension. Patients who received iloprost treatment for any indication within 48 h prior to enrollment in the clinical trial or patients who were on thrombin inhibitors or nitric oxide (NO) within the previous 24 h before study randomization were also excluded. Additionally, patients dependent on the sponsor, investigator, or their employees were not included in the study.

### Study drug

The IMP is iloprost (Ventavis®; Drug code SUB14185MIG; ATC code B01AC11), manufactured by Berlimed S.A., Madrid, Spain (for Bayer Pharma AG, Germany). It will be used as a concentrate for use in nebulizers and will be administered by inhalation three times a day (20 μg per administration). The administration of the drug will occur at the same time each day ± 1 h. In cases of severe adverse effects, the dosage will be reduced to 20 μg once a day (morning). Other dose modifications or temporary cessation of the study drug will not be allowed.

Iloprost is usually dissolved in 0.9% sodium chloride (NaCl), which is used to keep the ventilator circuit moist as standard of care. Therefore, in the control group, NaCl 0.9% will be used to keep the airway circuit moist, which is the standard of care for the treatment of patients with pulmonary insufficiency [7]. Considering the pharmacokinetic and dynamic profile of iloprost, we have suggested an approach of an application of three times per day, with a dose of 20 μg, which seems to be an average dose in the trials reported up to now. The rationale behind this was that iloprost also exerts an anti-inflammatory effect that may last up to 6 h [29–33]. Therefore, an administration of iloprost three times a day would allow a significant time frame per day to be covered by anti-inflammation due to this drug. The duration of 5 days was included in the trial because the pathophysiology of ARDS develops within the first few days and is progressive during that period.

### Randomization

Randomization lists will be generated at the biostatistical center. Based on these lists, numbered envelopes will be provided and used for randomization.

#### Concomitant medication and treatments

Relevant additional medications and treatments such as vasopressors, inotropes, anti-infective agents, inhalative therapy or sedation, steroids, and immunosuppressive therapy administered to the subjects on entry to the trial or at any time during the trial are regarded as concomitant medications and treatments and must be documented on the appropriate pages of the case report form (CRF); these data will be grouped according to class of medication. Depending on the substance, the documentation varies in details (e.g., dosing).

### Intervention plan

This study will consist of the following consecutive phases: study entry, treatment, and follow-up. The time points and trial procedures are listed in Table 2. All patients included in this trial will receive standard care for ARDS according to the ARDS network, with special consideration of lung-protective ventilation strategies.

**Table 2:** Table of events

Event	Presentation until start of iloprost or NaCl 0.9%	d1	d2	d3	d4	d5	d6–d27	Hospital discharge or d28	Hospital discharge or d90	d180 ± 14d
Informed consent	X									
Inclusion/exclusion criteria	X									
Pregnancy test in women of childbearing age	X									
Demographics	X									
Medical history	X									
Randomization	X									
Iloprost or NaCl 0.9% (control)		X	X	X	X	X				
Clinical assessment including outcome	X	X	X	X	X	X	X	X	X	
Laboratory testing	X	X	X	X	X	X	X	X		
Adverse/serious adverse event monitoring		X	X	X	X	X	X	X		
Plasma biomarkers	X	X	X	X	X	X				
Barthel Index	X							X	X	X
SOFA score	X	X	X	X	X	X	X	X		
Health-related questionnaire										X
VES										X

#### Study entry

In this trial, patients with ARDS present an emergency situation, such as the diagnosis of ARDS requiring intensive care unit (ICU) admission and ventilation therapy, which does not allow for any delay of diagnostic work-up or therapy. Additionally, due to severe symptoms, the vast majority of patients who meet the eligibility criteria for the trial are assumed to be unable to give consent in the acute admission phase, and legally authorized representatives (LARs) might not be available in most cases. This is also in line with local regulations: e.g., §41 of the German Drug Law allows the start of a treatment in an emergency situation without prior consent if the immediate treatment is necessary to save the patient’s life, recover the patient’s health, or ease the patient’s suffering. In this situation the consent of an independent physician not directly involved in the study conduct will be sought before the beginning of any study-related activity. The consent has to be obtained as soon as the patient is able to give consent or a LAR is available. Independently, personal consent will be obtained from each patient after recovering consciousness and competence for decision-making or by a legal representative in cases recovering is not achieved during the study duration (i.e., day 27). When possible, however, the patient or his legal representative is to be informed both in writing and verbally by the investigator before any study-specific procedure is performed. Each patient or his legal representative will be informed about the modalities of the clinical study in accordance with the provided patient information. Informed consent from the patient will be obtained using a form approved by the ethics committee (EC) of the Universitätsklinikum Tübingen or the local EC if the patient is treated in a collaborating institution.

#### Treatment phase

The treatment group will receive 20 μg of nebulized iloprost three times per day for 5 days in addition to standard care. Iloprost will be measured in blood samples to determine the serum levels within this setting.

**Fig. 1:**
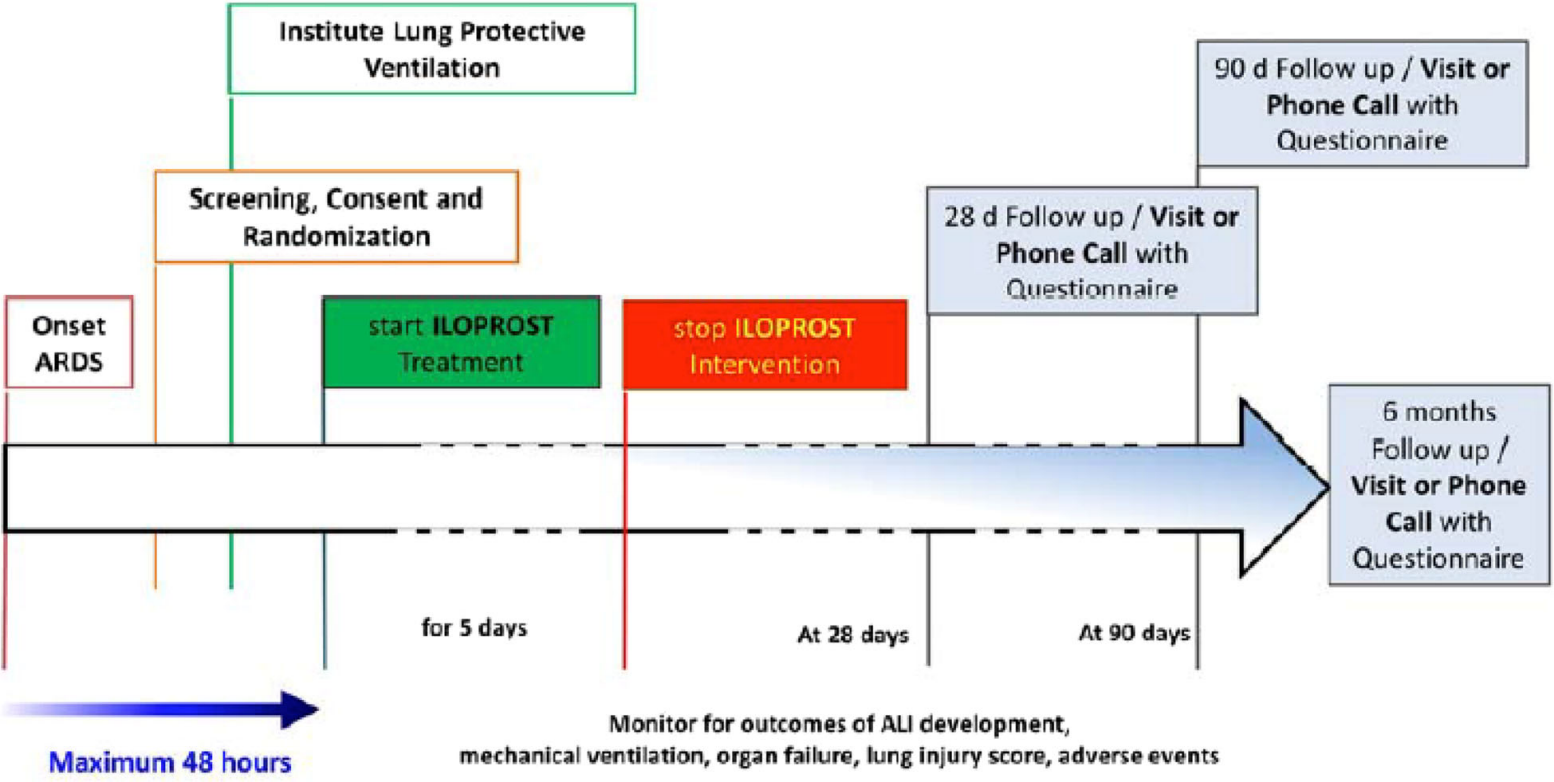
Trial protocol and intervention scheme. After screening and determination of eligibility, patients will be included after a maximum of 48 h after the onset of ARDS. Within this time period, screening, consent, and randomization will be initialized. In addition, lung-protective ventilation will be instituted. After randomization, iloprost 3 × 20 μg (intervention) or NaCl 0.9% (control) will be administered for 5 days through a standard ultrasound nebulizer. Daily recordings will be made with respect to the development of the PaO_2_/FiO_2_ ratio and the severity of ARDS, organ failure, lung injury, and potential adverse events. The treatment with iloprost or NaCl (0.9%) will be stopped after 5 days. The follow-up period will then continue up to 90 days and 6 months to determine the outcome, quality of life, and pulmonary/secondary organ function

The control group will receive nebulized 0.9% NaCl with an equal volume three times per day for 5 days. After 5 days, the trial treatment will be complete (Fig. 1). Blood samples will be drawn at defined time points for a variety of biomarkers to better assess the associations among coagulation, inflammation, and iloprost treatment. Key cointerventions (infection control, aspiration precautions, fluids, and transfusion) will be standardized across all patients. Mechanical ventilation will be standardized (see Additional file 1).

#### Follow-up

Hospital survivors will undergo a brief follow-up phone survey to assess functional status (Barthel Index), a health-related questionnaire, and the Vulnerable Elders Survey (VES) to assess frailty 6 months after enrollment. The patients will be visited daily until day 28 or until discharge from the ICU, which could be beyond day 28. If discharged, the next visit will be on day 90; if patients are still in the ICU, there will still be daily visits until this time point. Data will be collected according to the study procedure until then. Each visit will consist of a clinical examination, a blood sample, assessment of the functional capacity through the Barthel Index, and assessment of the severity of illness through the Sequential Organ Failure Assessment (SOFA) score. All data will be recorded on an electronic case report form (eCRF); this will be used as a visit diary. Blood samples will be drawn at defined visits for a variety of biomarkers to better assess the associations among coagulation, inflammation, and iloprost treatment (Table 3).

**Table 3:** Study assessments

	Admission	Iloprost administration								
		d1	d2	d3	d4	d5	ICU/ IMC	Ward	Hospital discharge or d28	Hospital discharge or d90
Laboratory testing										
Blood count	X^a^	X^a^	X^a^	X^a^	X^a^	X^a^	X^b^	X^c^	X	X
Procalcitonin	X^a^	X^a^	X^a^	X^a^	X^a^	X^a^	X^b^	X^c^	X	X
IL-6	X^a^	X^a^	X^a^	X^a^	X^a^	X^a^	X^b^	X^c^	X	X
PaO_2_/FiO_2_	X^a^	X^a^	X^a^	X^a^	X^a^	X^a^	X^b^	X^c^		
Hemoglobin	X^a^	X^a^	X^a^	X^a^	X^a^	X^a^	X^b^	X^c^	X	X
Hemostasis parameters	X^a^	X^a^	X^a^	X^a^	X^a^	X^a^	X^b^	X^c^	X	X
Renal parameters	X^a^	X^a^	X^a^	X^a^	X^a^	X^a^	X^b^	X^c^	X	X
Clinical parameters										
Ventilation support including ventilation parameters	X^a^	X^a^	X^a^	X^a^	X^a^	X^a^	X^a^			
Prone positioning	X^a^	X^a^	X^a^	X^a^	X^a^	X^a^	X^a^			
ECMO	X^a^	X^a^	X^a^	X^a^	X^a^	X^a^	X^a^			
Relaxation	X^a^	X^a^	X^a^	X^a^	X^a^	X^a^	X^a^			
High-frequency ventilation	X^a^	X^a^	X^a^	X^a^	X^a^	X^a^	X^a^			
Tracheotomy										
Hemodynamic parameters	X^a^	X^a^	X^a^	X^a^	X^a^	X^a^	X^b^	X	X	X
Vasopressor therapy	X^a^	X^a^	X^a^	X^a^	X^a^	X^a^	X^a^			
Inotrope therapy	X^a^	X^a^	X^a^	X^a^	X^a^	X^a^	X^a^			
Fluid balance	X^a^	X^a^	X^a^	X^a^	X^a^	X^a^	X^a^			
Transfusion of red blood cells	X	X	X	X	X	X	X^b^	X	X	X
Transfusion of thrombocytes	X	X	X	X	X	X	X^b^	X	X	X
Anticoagulation										
Infection	X	X	X	X	X	X	X^b^	X	X	X
Anti-infective therapy	X	X	X	X	X	X	X^b^	X	X	X
Length of stay in ICU										
Length of stay in hospital										
Discharge location									X	X
Death										
Cause of death										
Scores										
Richmond Agitation-Sedation Scale (RASS)	X^a^	X^a^	X^a^	X^a^	X^a^	X^a^	X^a^			
SOFA score^d^	X	X	X	X	X	X	X		X	X
Barthel Index									X	X

*IMC* intermediate care

^a^Assessment on daily basis during ICU stay

^b^Assessment on daily basis until day 14 and then once per week during ICU/IMC stay

^c^Assessment once per week on ward

^d^SOFA score during ventilation support once per week

^e^qSOFA score spontaneous breathing

### Outcome measurements

#### Study objectives

The primary objective and endpoint is to assess the effect of iloprost on the improvement of oxygenation (PaO_2_/FiO_2_ ratio) in patients with ARDS.

As secondary objectives, the absolute incidence of the following parameters will be determined:

- Overall survival in the 90-day follow-up period (90-day all-cause mortality)
- Duration of mechanical ventilation support
- ICU length of stay
- Ventilator-associated pneumonia
- Pulmonary hemorrhage
- Gastrointestinal hemorrhage
- Pulmonary embolism
- Delirium
- ICU-acquired weakness
- Discharge location (home, skilled nursing facility, rehabilitation).

The exploratory objectives are 6-month survival, quality of life (QOL) assessed with a short-form survey (SF12), functional status (Barthel Index), and frailty (VES) assessed by phone follow-up interview.

#### Efficacy parameters

The following parameters will be used to determine the treatment efficacy:

- Improvement of oxygenation (PaO_2_/FiO_2_) on a daily basis in relationship to baseline
- Overall survival in the 90-day follow-up period
- Decrease in duration and severity of ARDS
  - ◦ SOFA scores: to be calculated based on data in hospital records
  - ◦ Duration of mechanical ventilation support: documentation in hospital records
  - ◦ ICU length of stay: documentation in hospital records
  - ◦ Ventilator-associated pneumonia: documentation of microbiological findings in hospital records
  - ◦ Incidence of barotrauma: documentation of ventilator parameters in hospital records
- Reduced morbidity assessed through SOFA score, also according to the incidence of complications and increased functionality assessed through the Barthel Index
  - ◦ Delirium: documentation (e.g., confusion assessment method for the ICU [CAM-ICU]) in hospital records
  - ◦ ICU-acquired weakness: documentation in hospital records
  - ◦ Discharge location: documentation in hospital records, phone call.

The demographic parameters at enrollment include age, sex, race, ICU admission diagnosis, and comorbidities (such as diabetes, existing malignancy, any kind of pre-existing pulmonary disease, and hypertension).

The main clinical data obtained during the ICU daily assessment are as follows:

- Laboratory data: Blood count, procalcitonin, interleukin (IL)-6, creatinine, urea, partial thromboplastin time (PTT), D-dimers, international normalized ratio (INR), aspartate aminotransferase (AST), alanine aminotransferase (ALT), albumin, cholinesterase (CHE), brain natriuretic peptide (BNP)
- Ventilation support
  - ▪ Invasive or noninvasive ventilation
  - ▪ Prone positioning: Yes/No
  - ▪ Maximum P_max_ on daily basis
  - ▪ Maximum P_mean_ on daily basis
  - ▪ Minimum positive end-expiratory pressure (PEEP) on daily basis
  - ▪ Maximum PEEP on daily basis
  - ▪ Driving pressure at maximum P_max_
  - ▪ Maximum compliance on daily basis
  - ▪ Maximum FiO_2_ on daily basis
  - ▪ High-frequency ventilation: Yes/No
  - ▪ Tracheotomy: Yes/No
  - ▪ Any relaxation therapy performed: Yes/No
- Extracorporeal membrane oxygenation (ECMO) therapy: cannulation, blood flow, FiO_2_, sweep gas
- Volume resuscitation: volume crystalloids/day, volume colloids/day, volume albumin/day, daily balance sheet
- Transfusion: units red blood cells/day, units thrombocytes/day
- Substitution of clotting factors if necessary: fibrinogen, prothrombin complex concentrate (PPSB), factor XIII, tranexamic acid (Cyklokapron, Pfizer), von Willebrand factor (vWF), factor VIII, factor VII
- Anticoagulation: fractionated heparin (cumulative dose/day), unfractionated heparin (cumulative dose/day), epoprostenol (cumulative dose/day), argatroban (cumulative dose/day), others (cumulative dose/day)
- Hemodynamic parameters: heart rate, arrhythmia, lowest and highest mean arterial pressure (MAP) per day, lowest and highest central venous pressure (CVP) per day, cardiac index, lowest and highest SpO_2_ per day, lowest and highest lactate per day (mmol/L) SvO_2_
- Renal replacement therapy: Acute Kidney Injury Network (AKIN) criteria. Continuous renal replacement therapy (CRRT), continuous veno-venous hemodialysis (CVVHD), continuous veno-venous hemodiafiltration (CVVHDF), continuous veno-venous hemofiltration (CVVHF), intermittent dialysis, sustained low-efficiency dialysis (SLED)/dialysis, anticoagulation, duration, dose
- Delirium: CAM-ICU score
- Immunosuppressive therapy: Yes/No

Weekly assessments of the ICU will include the following:

- Differential blood count
- Creatinine clearance
- ECMO post-oxygenator PaO_2_
- SOFA score
- Assessment at discharge
- Chronic renal failure at discharge
- Hepatic failure at discharge
- Length of stay in the ICU
- Length of stay in the hospital
- Discharge from hospital to a nursing home
- Discharge from hospital to home
- Discharge from hospital to a rehabilitation unit
- Residence in nursing home at 6 months

The final assessment will consist of the following:

- Days of ECMO support
- Ventilator days
- Tracheotomy
- Need for mechanical ventilation at home
- Incidence of pulmonary hemorrhage defined by an indication for blood transfusion, radiological finding, or a decrease in oxygenation
- Incidence of barotrauma
- Incidence of pleural drainage
- Incidence of pulmonary embolism defined by the following parameters:
  - ◦ New hypotension
  - ◦ Sign of right ventricular failure on echocardiography
  - ◦ Biomarkers
  - ◦ Computed tomography (CT) scan (optional)
- Incidence of gastrointestinal bleeding defined by the following parameters: upper gastrointestinal bleeding, blood vomiting, lower gastrointestinal bleeding, melena, indication for blood transfusion, endoscopic diagnosis/intervention
- Incidence of cerebral hemorrhage defined by the following parameters: impairment as measured by the Glasgow Coma Scale, CT scan
- Infections: incidence of positive blood culture, pneumonia, wound infection, peritonitis, surgical intervention due to infection, bacterial infection, fungal infection, viral infection, or multidrug-resistant Gram-negative bacteria (MRGN) infection
- Anti-infective therapy: generic, duration, incidence of changing anti-infective therapy due to inadequate treatment
- Incidence of surgical intervention.

### Data collection and management

#### Case report form

The trial case report form (CRF) is the primary data collection instrument for the trial. For this project, electronic CRFs (eCRFs) will be used. Entered data will be subjected to plausibility checks directly implemented in the CRF, monitoring, and medical review. The trial master file, the CRFs, and other material supplied for the conduct of the study will be retained by the sponsor/clinical research organization (CRO) according to applicable regulations and laws. The investigator(s) will archive all trial data (source data and investigator site file [ISF], including the subject identification list and relevant correspondence) according to the International Conference on Harmonization of Technical Requirements for Registration of Pharmaceuticals for Human Use (ICH) consolidated guideline on good clinical practice (GCP) and local laws or regulations.

### Statistical analyses

#### Study population definition

The study population will consist of the following: those to be assessed for eligibility (*n* = 300); those to be assigned to the trial (*n* = 150); those to be analyzed (*n* = 150 in the intention-to-treat [ITT] analysis, other endpoints *n* = 120). The sample size and power consideration refers to 120 evaluable patients, and it is assumed that the power will not be decreased in the analysis of the ITT population using multiple imputation. Furthermore, baseline adjustment will not be taken into account, which leads to a conservative sample size estimation.

In a previous study on iloprost with 20 patients, an increase from 177 ± 60 to 213 ± 67 was observed for the PaO_2_/FiO_2_, which was significant at the 0.01 level [27]. Recalculation shows that the intraindividual standard deviation must have been considerably smaller, as a *p* value of 0.01 corresponds to an effect size of 0.93 (intraindividually) and thus to an intraindividual standard deviation of approximately 40 in this study.

In our study, we can show effect sizes of 0.525 assuming 116 error degrees of freedom, taking into account 1 day for baseline adjustment and 3 days for the study center (inquiry, power 80%, level of significance 0.05, two-sided *t* test). If we assume the recalculated standard deviation from the previous study in our study (which is still conservative due to the linear baseline adjustment used in our study), an (interindividual) effect size of 0.525 corresponds to a difference of approximately 21 in the PaO_2_/FiO_2_ ratio in the treatment arm compared to the control arm. This seems to be a reasonable and relevant effect.

#### Analysis of primary variables

The primary endpoint of PaO_2_/FiO_2_ at day 6 after the baseline will be analyzed daily using a baseline adjusted analysis of covariance model with the last measurement of the PaO_2_/FiO_2_ ratio before treatment as the baseline, with the study arm as a second-level factor. The study center will be included in the analysis as a nuisance factor. Additionally, an interaction term between baseline and treatment will be included in the model if this term is significant. In the case of interaction, the main effect will be retrieved for the arithmetic mean of the baseline values using the centered variable for PaO_2_/FiO_2_. Multiple imputation will be applied in the ITT population of patients receiving at least one dose of treatment or the control.

#### Analysis of secondary variables

Statistical analysis of the prespecified secondary endpoints will be performed with descriptive and exploratory statistical methods according to the scale and observed distribution (absolute and percentage frequencies, chi-square tests, logistic regression models for categorical variables; means and standard deviations, medians, and quartiles, or ranges with *t* tests or Mann-Whitney tests and linear regression models for continuous variables; Kaplan-Meier curves, log-rank tests, and Cox proportional hazard models for censored data). The *p* values will be reported but should not be considered part of the confirmatory analysis.

#### Subgroup analysis

Planned subgroup analyses will be performed according to the following:

- Sex and race (only for subgroups larger than 40 subjects)
- Patients with increased pulmonary arterial pressure
- Direct vs. indirect lung injury
- Age stratified by decades.

#### Stopping rules

For safety reasons, after the enrollment of 20 patients (day 28 after last dose IMP Patient 20), an interim analysis of the following will be performed:

1. An increased risk of pulmonary hemorrhage ≥ grade III according to CTCAE Version 5.0 in the treatment (iloprost) arm
2. Levels of IMP in the serum.

The results will be discussed with the DSMB. The DSMB has to assess whether the results allow continuation of the study as planned.

Moreover, after treatment of a total of 100 patients (day 28 after the last dose IMP Patient 100), an interim analysis of an increased risk of pulmonary hemorrhage ≥ grade III according to CTCAE Version 5.0 in the treatment (iloprost) arm will be performed, and the results will again be discussed with the DSMB. The DSMB must assess whether the results allow continuation of the study as planned.

Moreover, in the following situations, a premature termination of the trial must be considered:

3. Substantial changes in risk-benefit considerations
4. New insights from other trials
5. Insufficient recruitment rate.

#### Biometric report

The biometric report will be delivered according to the SOP BI07 of the statistical center (IKEaB). In summary, the report will contain sections on the statistical methodology, preprocessing of data, and the descriptive, exploratory, and confirmatory analyses. It will be reviewed by the principal investigator (PI) before presenting the final version.

## Discussion

To date, there is no pharmacologic intervention to treat or prevent the development of lung injury or ARDS. Iloprost-containing medications are well recognized epidemiologically as an effective therapeutic agent for the treatment of moderate to severe pulmonary hypertension. Iloprost has been shown to exert antiplatelet and anti-inflammatory actions in small clinical observation studies and several preclinical laboratory examinations. However, the use of iloprost for the treatment of ARDS is not novel; it has been used in small studies before. Indeed, we propose in this study to systemically evaluate the application of iloprost in a randomized controlled trial (RCT) to identify the potential use and benefit of iloprost in ARDS. The composite endpoint was chosen, as it is likely to be more sensitive than just 28-day mortality to detect an effect signal. Although it is not a double-blinded strategy, the recorded objectives will help support or refute our hypothesis that iloprost reduces lung inflammation during early ARDS.

This study includes some possible pitfalls, like the single-blinded design. However, due to randomization and based on the close data acquisition, we will be able to minimize bias.

However, in addition to the effect of iloprost on lung inflammation, this study will also be a resource for information about clotting issues in terms of the systemic and local anticoagulation effects of iloprost in lung tissue, and also in other compartments besides the lung. Although iloprost is used frequently in pulmonary hypertension, there are currently no data about iloprost concentration in the blood after inhalative treatment. In addition, iloprost may have a positive effect on lung compliance during acute ARDS as well as during resolution, since it has been shown to have a lasting positive effect on fibrosis in the lung and other tissues in animal models [34, 35]. In one-lung ventilation, iloprost seems to reduce intrapulmonary shunts, resulting in better oxygenation [36, 37]. In this context, the analysis of ventilator-free days or time on ECMO may reveal important information. Further on, intravenous application of iloprost may improve microcirculation, resulting in better kidney recovery in patients with sepsis [37, 38]. Patients with ARDS frequently show multiorgan failure. Therefore, the comparison of incidence and time frame for extracorporeal therapy may give insights on the effect of inhaled iloprost on microcirculation in other organs. Therefore, iloprost may positively influence the outcome of ARDS patients by at least one of the effects described above. This study will be the first to describe the effects of iloprost on inflammation, fibrosis, bleeding events, and oxygenation organ failure and anticoagulation during a continuous time frame of at least 5 days in critically ill patients.

### Trial status

Patient recruitment started on 5 July 2019 based on the 4th version of the protocol released 22 May 2019 and approved by the ethics committee of the University of Tübingen, Germany (899/2018AMG1) on 4 June 2019. The recruitment will be finished approximately in July 2021.

## Supplementary information

**Additional file 1.** Ventilation management.

**Additional file 2.** Standard Protocol Items: Recommendations for Interventional Trials (SPIRIT) checklist.

## Data Availability

The datasets used and/or analyzed during the current study are available from the corresponding author on reasonable request.

## Abbreviations

ADR: Adverse drug reaction
AE: Adverse event
AKIN: Acute Kidney Injury Network
ALI: Acute lung injury
ALT: Alanine aminotransferase
ARDS: Acute respiratory distress syndrome
AST: Aspartate aminotransferase
BfArM: Bundesinstitut für Arzneimittel und Medizinprodukte
BNP: Brain natriuretic peptide
CAM-ICU: Confusion assessment method for the ICU
CD11b: Fibrinogen receptor activation
CHE: Cholinesterase
COX-2: Cyclooxygenase-2
CRF: Case report form
CRRT: Continuous renal replacement therapy
CT: Computed tomography
CTCAE: Common Terminology (Toxicity) Criteria for Adverse Events
CVVHD: Continuous veno-venous hemodialysis
CVVHDF: Continuous veno-venous hemodiafiltration
CVVHF: Continuous veno-venous hemofiltration
DAMP: Damage-associated molecular pattern
DFG: Deutsche Forschungsgemeinschaft
eCRF: Electronic case report form
FiO_2_: Inspiratory oxygen concentration
GCP: Good clinical practice
HFO: High-frequency oscillation
IC: Informed consent
ICAM-1: Intercellular adhesion molecule 1
ICH: International Conference on Harmonization of Technical Requirements for Registration of Pharmaceuticals for Human Use
ICU: Intensive care unit
IFNγ: Interferon-γ
IL-6: Interleukin-6
IMC: Intermediate care
IMP: Investigational medicinal product
INR: International normalized ratio
IR: Ischemia-reperfusion (injury)
ISF: Investigator site file
ITT: Intention to treat
MAP: Mean arterial pressure
NET: Neutrophil extracellular trap
NO: Nitric oxide
PAH: Pulmonary arterial hypertension
PAMP: Pathogen-associated molecular pattern
PaO_2_: Partial pressure of oxygen
PEEP: Positive end-expiratory pressure
PNC: Platelet-neutrophil complexes
PPSB: Prothrombin complex concentrate
PRR: Pattern recognition receptor
P-selectin: Platelet activation factor P-selectin
PTT: Partial thromboplastin time
qSOFA: Quick SOFA
RAP-1: Ras-related protein 1
RASS: Richmond Agitation-Sedation Scale
RCT: Randomized controlled trial
SAE: Serious adverse event
SLED: Sustained low-efficiency dialysis
SOFA: Sequential Organ Failure Assessment (score)
SpO_2_: Oxygen saturation
SvO_2_: Central venous oxygen saturation
TNFα: Tumor necrosis factor-α
VASP-P: Phosphorylation of vasodilator phosphoprotein
VCAM-1: Vascular adhesion molecule 1
VES: Vulnerable Elders Survey
vWF: von Willebrand factor
CVP: Central venous pressure

## References

[1] Ferguson ND, Fan E, Camporota L, Antonelli M, Anzueto A, Beale R (2012). The Berlin definition of ARDS: an expanded rationale, justification, and supplementary material. Intensive Care Med.

[2] Bernard GR, Artigas A, Brigham KL, Carlet J, Falke K, Hudson L (1994). The American-European Consensus Conference on ARDS. Definitions, mechanisms, relevant outcomes, and clinical trial coordination. Am J Respir Crit Care Med.

[3] Lee CM, Hudson LD (2001). Long-term outcomes after ARDS. Semin Respir Crit Care Med.

[4] Neff TA, Stocker R, Frey HR, Stein S, Russi EW (2003). Long-term assessment of lung function in survivors of severe ARDS. Chest..

[5] Matthay MA, Ware LB, Zimmerman GA (2012). The acute respiratory distress syndrome. J Clin Invest.

[6] Ware LB, Matthay MA (2000). The acute respiratory distress syndrome. N Engl J Med.

[7] (AWMF) AdWMFeV. Leitlinie Invasive Beatmung und Einsatz extrakorporaler Verfahren bei akuter respiratorischer Insuffizienz 2017; AWMF Leitlinien-Register Nr. 001/021; 2017. https://www.awmf.org/uploads/tx_szleitlinien/001-021l_S3_Invasive_Beatmung_2017-12.pdf.

[8] Patel BK, Wolfe KS, Pohlman AS, Hall JB, Kress JP (2016). Effect of noninvasive ventilation delivered by helmet vs face mask on the rate of endotracheal intubation in patients with acute respiratory distress syndrome: a randomized clinical trial. JAMA.

[9] Gattinoni L, Carlesso E, Taccone P, Polli F, Guerin C, Mancebo J (2010). Prone positioning improves survival in severe ARDS: a pathophysiologic review and individual patient meta-analysis. Minerva Anestesiol.

[10] Kor DJ, Carter RE, Park PK, Festic E, Banner-Goodspeed VM, Hinds R (2016). Effect of aspirin on development of ARDS in at-risk patients presenting to the emergency department: the LIPS-A randomized clinical trial. JAMA.

[11] McAuley DF, Laffey JG, O'Kane CM, Perkins GD, Mullan B, Trinder TJ (2014). Simvastatin in the acute respiratory distress syndrome. N Engl J Med.

[12] National Heart L, Truwit JD, Bernard GR, Steingrub J, Matthay MA, Blood Institute ACTN (2014). Rosuvastatin for sepsis-associated acute respiratory distress syndrome. N Engl J Med.

[13] Birukova AA, Wu T, Tian Y, Meliton A, Sarich N, Tian X (2013). Iloprost improves endothelial barrier function in lipopolysaccharide-induced lung injury. Eur Respir J.

[14] Malmros C, Blomquist S, Martensson L, Thorne J (1994). Iloprost attenuates trauma-related pulmonary sequestration of leucocytes and platelets. Prostaglandins Leukot Essent Fatty Acids.

[15] Birukova AA, Fu P, Xing J, Cokic I, Birukov KG (2010). Lung endothelial barrier protection by iloprost in the 2-hit models of ventilator-induced lung injury (VILI) involves inhibition of Rho signaling. Transl Res.

[16] Scully M, Gang C, Condron C, Bouchier-Hayes D, Cunningham AJ (2012). Protective role of cyclooxygenase (COX)-2 in experimental lung injury: evidence of a lipoxin A4-mediated effect. J Surg Res.

[17] Birukova AA, Fu P, Xing J, Birukov KG (2009). Rap1 mediates protective effects of iloprost against ventilator-induced lung injury. J Appl Physiol (1985).

[18] Erer D, Dursun AD, Oktar GL, Iriz E, Zor MH, Elmas C (2014). The effects of iloprost on lung injury induced by skeletal muscle ischemia-reperfusion. Bratisl Lek Listy.

[19] Kawashima M, Nakamura T, Schneider S, Vollmar B, Lausberg HF, Bauer M (2003). Iloprost ameliorates post-ischemic lung reperfusion injury and maintains an appropriate pulmonary ET-1 balance. J Heart Lung Transplant.

[20] Yasa H, Yakut N, Emrecan B, Ergunes K, Ortac R, Karahan N (2008). Protective effects of levosimendan and iloprost on lung injury induced by limb ischemia-reperfusion: a rabbit model. J Surg Res.

[21] Erer D, Ozer A, Demirtas H, Gonul II, Kara H, Arpaci H (2016). Effects of alprostadil and iloprost on renal, lung, and skeletal muscle injury following hindlimb ischemia-reperfusion injury in rats. Drug Des Devel Ther.

[22] Wittwer T, Franke UF, Fehrenbach A, Ochs M, Sandhaus T, Schuette A (2005). Donor pretreatment using the aerosolized prostacyclin analogue iloprost optimizes post-ischemic function of non-heart beating donor lungs. J Heart Lung Transplant.

[23] Wittwer T, Franke UF, Ochs M, Sandhaus T, Schuette A, Richter S (2005). Inhalative pre-treatment of donor lungs using the aerosolized prostacyclin analog iloprost ameliorates reperfusion injury. J Heart Lung Transplant.

[24] Dembinski R, Brackhahn W, Henzler D, Rott A, Bensberg R, Kuhlen R (2005). Cardiopulmonary effects of iloprost in experimental acute lung injury. Eur Respir J.

[25] Wittwer T, Franke UF, Sandhaus T, Thiene M, Groetzner J, Strauch JT (2007). Preischemic iloprost application for improvement of graft preservation: which route is superior in experimental pig lung transplantation: inhaled or intravenous?. Transplant Proc.

[26] Wittwer T, Pethig K, Struber M, Hoeper M, Harringer W, Haverich A (2001). Aerosolized iloprost for severe pulmonary hypertension as a bridge to heart transplantation. Ann Thorac Surg.

[27] Sawheny E, Ellis AL, Kinasewitz GT (2013). Iloprost improves gas exchange in patients with pulmonary hypertension and ARDS. Chest.

[28] Afshari A, Bastholm Bille A, Allingstrup M (2017). Aerosolized prostacyclins for acute respiratory distress syndrome (ARDS). Cochrane Database Syst Rev.

[29] Guneysu E, Kocman AE, Ozatik O, Ovali C, Can B, Alatas IO (2017). The effects of iloprost and alprostadil on ischemia-reperfusion injury in preventing inflammation, tissue degeneration, and apoptosis in rat skeletal muscle. Turk J Med Sci.

[30] Rose F, Hattar K, Gakisch S, Grimminger F, Olschewski H, Seeger W (2003). Increased neutrophil mediator release in patients with pulmonary hypertension—suppression by inhaled iloprost. Thromb Haemost.

[31] Opitz CF, Wensel R, Bettmann M, Schaffarczyk R, Linscheid M, Hetzer R (2003). Assessment of the vasodilator response in primary pulmonary hypertension. Comparing prostacyclin and iloprost administered by either infusion or inhalation. Eur Heart J.

[32] Krause W, Krais T (1986). Pharmacokinetics and pharmacodynamics of the prostacyclin analogue iloprost in man. Eur J Clin Pharmacol.

[33] Yilmaz Y, Tumkaya L (2019). Effects of hyperbaric oxygen and iloprost on intestinal ischemia-reperfusion induced acute lung injury. Ann Surg Treat Res.

[34] Gomez-Arroyo J, Sakagami M, Syed AA, Farkas L, Van Tassell B, Kraskauskas D (2015). Iloprost reverses established fibrosis in experimental right ventricular failure. Eur Respir J.

[35] Aytemur ZA, Hacievliyagil SS, Iraz M, Samdanci E, Ozerol E, Kuku I (2012). Effects of iloprost on bleomycin-induced pulmonary fibrosis in rats compared with methyl-prednisolone. Rev Port Pneumol.

[36] Choi H, Jeon J, Huh J, Koo J, Yang S, Hwang W (2019). The effects of iloprost on oxygenation during one-lung ventilation for lung surgery: a randomized controlled trial. J Clin Med.

[37] Johannes T, Ince C, Klingel K, Unertl KE, Mik EG (2009). Iloprost preserves renal oxygenation and restores kidney function in endotoxemia-related acute renal failure in the rat. Crit Care Med.

[38] Depret F, Sitbon A, Soussi S, De Tymowski C, Blet A, Fratani A (2018). Intravenous iloprost to recruit the microcirculation in septic shock patients?. Intensive Care Med.

