# Supplementary material for: Therapeutic iloprost for the treatment of acute respiratory distress syndrome (ARDS) (the ThIlo trial): a prospective, randomized, multicenter phase II study
Source: Trials. 2020 Mar 4;21:242. doi: 10.1186/s13063-020-4163-0 (PMC7057516; doi:10.1186/s13063-020-4163-0)
Supplement: Supplementary file 1 — Additional file 1. Ventilation management. [file 13063_2020_4163_MOESM1_ESM.docx]

**Additional file 1**

**Ventilation settings and management**

In this study, the ventilation settings are based on the S3 Guideline Invasive Ventilation (AWMF Register # 001 - 021) and the recommendations of the ARDS Network.

1. Any type of ventilation that allows you to set an ideal tidal volume (VT) can be used. The VT target (6 ml / kgKG +/- 2 ml / kgKG) must be monitored. When the APRV ventilation mode is used, the tidal volume is defined as the average spontaneous VT estimate.
2. VT target is 6 ml / kgKG +/- 2 ml / kgKG
3. Plateau inspiratory pressure (Pplat) should be documented according to the ICU routine at least every four hours and / or after changes in VT and PEEP
4. If Pplat > 30 cmH2O, then the VT should be reduced to 5 ml / kgKG and if necessary to 4 ml / kgKG to keep Pplat ≤ 30 cmH2O.
5. If the platelet is <25 cmH2O, VT should be increased by 1 ml / kg until the VT target is reached
   The PEEP minimum is 5 cmH2O
   The maximum ventilation rate should be 35 / minute, the pH should be> 7.3.
   When setting the I: E ventilation conditions ideally I≤E should be followed.
   Several ventilation settings can be changed at the same time, as far as medically necessary.

Please note: Crucial are the settings on the ventilator.

PEEP settings accoording to ARDS-Network

The following tables provide help according to the ARDS-Network for setting FiO_2_ and PEEP.

**Low PEEP/High FiO_2_ group**

| FiO_2_ | .30 | .40 | .40 | .50 | .50 | .60 | .70 | .70 | .70 | .80 | .90 | .90 | .90 | 1.0 |
| --- | --- | --- | --- | --- | --- | --- | --- | --- | --- | --- | --- | --- | --- | --- |
| PEEP (cmH_2_O) | 5 | 5 | 8 | 8 | 10 | 10 | 10 | 12 | 14 | 14 | 14 | 16 | 18 | 18-24 |

**High PEEP/Low FiO_2_ group**

| FiO_2_ | .30 | .30 | .30 | .30 | .30 | .40 | .40 | .50 | .50 | .50 – .80 | .80 | .90 | 1.0 | 1.0 |
| --- | --- | --- | --- | --- | --- | --- | --- | --- | --- | --- | --- | --- | --- | --- |
| PEEP (cmH_2_O) | 5 | 8 | 10 | 12 | 14 | 14 | 16 | 16 | 18 | 20 | 22 | 22 | 22 | 24 |
